# Laughter as a Health Promoter

**Published:** 1890-02

**Authors:** 


### LAUGHTER AS A HEALTH PROMOTER.

In the "Problem of Health," Dr. Greene says that there is not the remotest corner or little inlet of the minute blood vessels of the human body that does not feel some wavelet from the convulsions occasioned by good hearty laughter. The life principle of the central man is shaken to its innermost depths, sending new tides of life and strength to the surface, thus materially tending to insure good health to the persons who indulge therein. The blood moves more rapidly, and conveys a different impression to all the organs of the body, as it visits them on that particular mystic jonrney when the man is laughing, from what it does at other times. For this reason every good hearty laugh in which a person indulges tends to lengthen his life, conveying, as it does, new and distinct stimulus to the vital forces.—*London Standard.*

